# A Practical Treatise on Midwifery

**Published:** 1844-10-01

**Authors:** 


### A Practical Treatise on Midwifery. By *W. Chailly.* Translated from the French, and edited by *G. S. Bedford,* M.D. New York, 1844. Pp. 528.

It is to the grateful enthusiasm which Dr. Bedford feels towards the teachers of the French schools, rather than to the intrinsic merits of the publication, that we owe this translation. The great lying-in hospital of Paris, and the system of clinical instruction there practised, (conducted however with a useless indecency which would be tolerated by no class of females, however degraded, among ourselves,) certainly offer greater advantages to the pupil's speedy acquisition of a knowledge of some of the difficulties of this branch of the profession, than our own institutions, with the exception of the Dublin Lying-in Hospital, do. But, if we mistake not, by this great accumulation of difficult cases within a brief space of time, the student is sometimes misled into a false estimate of their true proportion; and, at the same time that he acquires greater facility in employing manual interference, is apt to be impressed with the idea that such is much more frequently necessary than it really is. We all know how the extraordinary cases fix the attention of the hospital students, so that their teachers are frequently obliged to remind them that few of these in after-life will fall to their lot. The plan adopted in this metropolis of attending the patients at their own homes, has at least the advantage of exhibiting practice to the student as it really exists; and, in this and our other large towns, the opportunity of attending as many cases as may be desired or necessary is never wanting, while the advice and assistance of senior pupils, and of the teachers themselves, are always forthcoming at the desired moment. Testing each by the results, at least, we have no cause for dissatisfaction; for, whether we consider the actual condition of the practice of midwifery in this country, or the sterling character of the vast number of scientific and practical works which have been written for its illustration, we feel conscious of a proud pre-eminence. This is no idle boast, and if we were disposed to be vain-glorious, this work itself would furnish us with the material; bearing testimony, as it does, to the superiority of the English practice, in more than one important point, by adopting it, and that too in the very cases in which it has been so long the fashion in France to decry the same. Thanks to the labours of Denman, Hunter, and their successors, that which was looked upon heretofore as a low art, or, at all events, as a very subordinate branch of the profession, has been elevated during the last half-century to the dignity of a scientific pursuit, capable of giving employment to the most profound research, and to call forth the powers of the highest genius. Nor has such elevation been accomplished by the aid of either of the great medical corporate bodies, who have acted indeed rather the parts of cruel step-mothers than of tender foster-parents; and although one of these, struck perhaps by the comely appearance of her neglected child, has thought proper to acknowledge the error, and receive her to her arms, the other shows at present anything but signs of compunction.

We were not surprized to observe, during the perusal of this work, little or no reference to the authors of our own country, so that a French student rises from its perusal unacquainted with even the names of those who have contributed so much to the modern advancement of midwifery. But, however consistent this is with the usual practice of French medical writers, we must confess we have felt much surprise that Dr. Bedford has in no-wise supplied the deficiency. He states, in the title-page, that he has not only translated but *edited* the work; and, although we do not understand the sense in which he employs the last word, it seems to us, occupying as he does the post of professor of midwifery, the sense in which he ought to have employed it should have been in furnishing information to his pupils of the sources whence the deficiencies of the author might be supplied from. He attempts nothing of the kind, but seems to think the whole matter of the obstetrical art and science is comprised in this very imperfect work.

Having said thus much upon the incompleteness of Dr. Chailly's treatise, we may observe that some parts are well executed, especially all that relates to delivery, and manual interference; and, although the author still adheres to practices with the forceps which we consider reprehensible, he has manifestly advanced farther in the career of improvement, than any continental writer with whom we are acquainted.

We proceed to make a few extracts.

### Premature Artificial Delivery and the Cæsarean Section.

This chapter should be printed in letters of gold, as showing the triumph over prejudices we had began to think were inveterate. It is somewhat humiliating for France that her practitioners should have so long consented to obey the mandates of priestly dictators; and, that, long after that incubus had been removed, they should have been consulting about, protesting against, and stigmatizing others for pursuing, a practice which common sense and humanity dictate, and which all practitioners of eminence in any other part of Europe have been unanimous in recommending. But, however, *nous avons changé tout cela,* and M. Chailly is as determined an advocate of the proceeding as can be desired.

> "Thanks to the efforts of MM. Stoltz, Dezeimeris, Dubois, and Velpeau, delivery brought on before the full time is an operation hereafter recognized in French midwifery. For a long time it proved useful to our neighbours in England and Germany, while a foolish prejudice caused it to be rejected by French practitioners, who do not hesitate to have recourse even to the Cæsarean section and symphyseotomy. If we consult the statistics published in 1838 by M. Stoltz, it will be seen that, in 211 premature artificial deliveries, more than one-half the children survived, and scarcely one woman in 15 died: now, compare these results with those obtained from the Cæsarean Section and Symphyseotomy, and then decide. We have not within the walls of Paris one solitary example of a woman who has survived the Cæsarean Section. She who lived the longest was one of those on whom I assisted M. Dubois to operate. She died on the 17th day of a tetanic affection, when everything promised a most successful result." 131.

And again, when treating specially of the *Cæsarean Section,* in another part of his work, the author observes—

"The Cæsarean operation is one of the most serious that can be practised on the living woman; *five-sixths, at least, die.* With such results, it is difficult to conceive how a practitioner can decide to perform it, when, by sacrificing the child, it is possible to save the mother. Thus, when the pelvis measures more than two inches, cephalotripsy should be had recourse to. It will be in vain objected that the use of the cephalotribe (a compressing forceps invented by Baudelocque, jun.) is murderous, when the pelvis measures but a trifle over two inches. To this it may be replied, that this instrument, even in these conditions, presents a much better chance of life to the mother than the Cæsarean Section; and if the cephalotribe has not yet rendered all the service it will one day show itself capable of doing, it is because accoucheurs are not sufficiently familiar with its use, and because, in most cases, it is not employed until the female, already exhausted by a prolonged labour, and by attempts of every description to relieve her, is about expiring. While the Cæsarean operation, on the contrary, is, and should always be, performed under favourable circumstances, such as will rarely be absent when the fœtus is living.

"I am fully of opinion, that the period will arrive when the generation which succeeds us, judging without prejudice, will render proper justice to the cephalotribe, and proscribe the Cæsarean Section in cases in which, by destroying the fœtus, it will be possible to save the mother. At present I will not practise the abdominal and uterine section, in order to extract the fœtus, except when the pelvis is so contracted as to render the mutilation of the fœtus impossible. But when the contraction is less than two inches, the Cæsarean operation must be resorted to; this is the only resource left to the mother; and, if the operation be performed at the proper time, the child may be saved."

The author objects even to the indiscriminate performance of the operation even after the death of the mother.

"If the female should succumb during labour, it would be better, if the condition of the organs would permit, to extract the fœtus by means of the forceps, or by version. How often, after the death of the mother, has this operation been practised; and practised, too, even when auscultation has not detected the pulsations of the fœtal heart? Some accoucheurs recommend always to perform it, whether the pulsations of the heart are heard or not, and even after the lapse of 10, 15, or 24 hours from the death of the mother, children having survived the death of their parent for this period. There can be no objection to this precept as a general rule of conduct, being always careful to ascertain that the female has really expired, and careful too to perform the operation with as much vigilance as if she were living, for fear that she really were so. When, however, this rule was originally laid down, its utility was undoubted, for at that period there were no certain signs by which the life or death of the child could be established. But we are now perhaps not so well convinced of its necessity, since auscultation furnishes the certain means of ascertaining the pulsations of the heart: a means the more infallible, as all the other organs in which life is extinct are in the most absolute repose, so as not in any way to interfere with auscultation. As for myself, I do not hesitate to state, that, unless the friends should urgently desire the operation, (nor yet if they did we should suppose,) I would not perform it, unless I had distinctly heard the pulsations of the fœtal heart. And I should pursue this course for the reason that the death of the mother might be only apparent; and if the operation be performed only 10 or 15 minutes after it was supposed she had expired, her death would certainly be caused on the spot, while, without the operation, she might have been restored to life." 388.

These statements of M. Chailly become invested with great importance when we recollect, that he is delivering the sentiments of the medical officers of the chief midwifery institution in France; and that, "after the deliberation of the Royal Council, the use of this Treatise is authorized in the Faculties, in the Schools of Medicine, and in the different courses instituted for the instruction of sage-femmes, Villemain, Minister of Public Instruction."

May we not hope that, in the course of another half-century, some writer on French Clinical Midwifery may be able to apply to Paris the observation made by Dr. Robert Lee, in reference to London:—"There is no eminent accoucheur now practising in London, who has been present at the performance of the operation upon the living body, or who would recommend it, if delivery could be effected by the perforator and crotchet."

The American translator, however, is disposed to think M. Chailly is too hasty in his abandonment of the Cæsarean Section. He says, in a note to the above—

> "The Cæsarean Section is, undoubtedly, a dread alternative for the accoucheur to choose, but I cannot agree with Dr. Chailly that its fatality is as great as he represents; nor am I disposed to adopt the opinion, unfortunately too general, that craniotomy is almost always to be preferred to the Cæsarean Section. In truth, it needs some nerve, and for a man of high moral feeling, much evidence as to the necessity of the operation, before he can bring himself to the perpetration of an act which requires, for his own peace of mind, the fullest justification. The man who would wantonly thrust an instrument of death into the brain of a living fœtus, would not scruple, under the mantle of night, to use the stiletto of the assassin. Yet, how often has the fœtus been recklessly torn from its mother's womb piece-meal, and its fragments held up to the contemplation of the astonished and ignorant spectators as testimony undoubted of the operator's skill. Oh! could the grave speak, how eloquent, how momentous, how damning to the character of those who speculate in human life, would be its revelations!"

We are not disposed to denounce the use of the strongest language by the lecturer to his pupils in order to deter them from the rash use of instruments, especially those of a destructive character; and we have no doubt, from facts incidentally mentioned in the notes of this work, such is much required in the editor's country; but we submit that, in the present case, it is a little misplaced. The cases under discussion admit of no doubt as to the necessity of interference of some kind, and the only question is, whether the child or the mother is to be sacrificed; or, at the very least, whether the life of the latter is to be placed in great additional jeopardy, for the sake of attempting to save that of the former. Little art would be required to produce a picture as graphic of the horrors accompanying the Cæsarean Section as Dr. Bedford has drawn of those attending embryotomy—horrors, be it remembered, perpetrated upon a being whose sensibilities are worked up to the utmost agony, and whose importance, if not to society, at least to her family, is often inappreciable; and whose suffering, and value of whose life can scarcely be compared with that of her child, who, if not dead already, is often dead to sensation by reason of the long-continued pressure upon its brain, and has contracted no duties, ties, or sensations, as has a living being.

Dr. Bedford objects that the mortality from the Cæsarean Section has been exaggerated. Referring to Dr. Churchill's Tables, he finds that about one mother in five has died after craniotomy, and one out of $2\frac{1}{2}$ recovered after the Cæsarean Section. In answer to this we would state, that the very horror which most practitioners feel for performing craniotomy too often induces them to *delay the operation* so long that the mother's powers have become exhausted beyond the power of revival. There is nothing in the nature of the operation itself to lead to so fatal a result. The case is very different as regards the Cæsarean, in which large and important organs are wounded. Moreover, in regard to this last, we are more disposed to attach importance to Dr. Chailly's statements, that five-sixths die after it, than to Dr. Churchill's Tables, which comprise so insignificant a number of cases: viz. 321 since 1750. These, compared with the number which have occurred on the Continent, and have never been recorded, are as nothing. The real proportionate mortality can therefore never be accurately ascertained, and is much more likely to be approximated by one living in a country where the operation has been so frequently undertaken, than by the mere compiler, however able, of published cases.

Dr. Bedford refers to a remarkable case in which the subject of it had so great a deformity of the pelvis, that the antero-posterior diameter did not exceed two inches. In her first labour, 1831, embryotomy was performed, as also in her second, in 1833. In 1835 and 1837, this "heroic woman" underwent the Cæsarean section, mother and child on each occasion doing well. He also relates another case of a dwarf, in whom the Cæsarean section was performed with perfect success—the pelvic diameter not exceeding $1\frac{1}{2}$ inch. He considers this operation should always be resorted to when the antero-posterior diameter of the superior strait measures less than two inches, on account of the hazard of laceration the mother incurs. "My opinion upon this subject is not a speculative one: it is based on repeated examination made on a wooden pelvis constructed for this purpose; and I am satisfied that even with the space of $2\frac{1}{4}$ inch, all the dexterity which the operator can bring to his aid will be required to protect the mother from serious, if not fatal injury."

### The Forceps.

M. Chailly simplifies much some of the rules and proceedings of his predecessors; but he, like all other French writers, recommends the application of this instrument in cases in which English practitioners consider craniotomy the best practice, especially when the head is high up above the brim, or has been impacted for an excessively long period. With us the guiding point is the safety of the mother, not neglecting that of the child where attending to it will not compromise that of its parent. There can be no doubt that, when not delayed too long, for this is a principal cause of death from labour requiring interference, the embryotomy instruments may be employed with far less risk of injuring the mother than can the forceps. This cautious use of the forceps in Britain has given rise to the laying down a rule severely commented upon by the translator of the present work, but yet of whose utility we feel convinced. Dr. Bedford goes the whole length of the French practice, and, after quoting passages from the work of Dr. Ramsbotham (as one of our latest authors), in which the necessity of being able to feel one or both of the *ears of the child* before applying the forceps is insisted upon, thus proceeds:—

"In the first place, I would observe, that my own experience teaches me that it is not an easy thing to reach the ear, even when the head is at the inferior strait, and, secondly, if the rule, as laid down by Dr. R. be adopted, fatal consequences must inevitably frequently ensue to both mother and child.

"To illustrate this point, let us suppose that the head is in the pelvic cavity; the mother suddenly becomes exhausted, either from hæmorrhage, or the fatigue of antecedent effort. No matter what the cause may be, she is exhausted, and immediate delivery is indicated. The accoucheur introduces the finger, and endeavours to reach the ear; he does not succeed; the patient's situation becomes more and more alarming; he again makes the attempt to find the ear; he fails; he feels in his heart, indeed every thing clearly indicates, that the forceps should be applied, but '*he cannot reach the ear:*' he delays, in the hope that '*the head may come down into the pelvis sufficiently low to enable him to feel one or both ears distinctly.*' Alas! this proves fallacious. The assistants supplicate him to do something to relieve the patient, for they see she is dying; and what will it avail, under these melancholy circumstances, for him to exclaim 'I can do nothing, *for the ear of the child cannot be felt.*' His patient of course, sinks, and here are two lives sacrificed because of a precept in which I can see neither propriety or meaning. Let it not be supposed that this is an overdrawn picture. Such results must inevitably ensue from an adherence to the rule to which I have alluded. When Dr. Ramsbotham says '*that it is necessary to touch one or both ears, because they become the guide to the proper adaptation of the blades,*' he makes use of language that, I must confess, surprises me not a little. If there be any meaning in what he says, it is simply this, that unless the ears are felt, it will be impossible to arrange the blades of the forceps, because of the ignorance of the accoucheur as to the position of the head. Admitting the truth of this author's reasoning, when the head is at the inferior strait, which I most unequivocally deny, how is the position to be ascertained when the head is still at the superior strait? Certainly not by feeling the ears, for these cannot be felt once in a thousand times, before the head has descended into the pelvic cavity. The position of the head can be told both at the inferior and at the superior strait by the direction of the fontanelles, sagittal suture, &c. &c.; and these will indicate the manner of applying the forceps, and of seizing the head in its bi-parietal measurement.

"The rule therefore for the pupil to adopt, is to pay no regard either to the ear or the length of time the head may have been in the excavation (!), but to proceed to artificial delivery the moment the life of either mother or child becomes seriously endangered. The very essence of forceps' delivery, that which commends it so strongly to the consideration of the profession, is the ability with which it enables us to save both mother and child. Therefore, if artificial delivery be indicated, *have recourse to it before the life of the child has been sacrificed, or the vital force of the mother so far expended as to render her recovery extremely doubtful.* I do not advocate a meddlesome midwifery, but I do most strenuously recommend such an application of the means put into our hands of affording relief as will achieve the minimum of good to both mother and child." 316.

The case put by Dr. Bedford of hæmorrhage or other complication of labour rendering speedy delivery a paramount object, the pelvis being as he supposes natural and proportionate to the size of the child, may be easily disposed of. If the head had entered the pelvis, the ears could assuredly be felt, and the forceps or vectis would be applied by every practitioner. If, on the contrary, it had not, version would be resorted to, and with far less chance of injury to the mother, a far more speedy termination of her sufferings, and less risk to the life of the child, than could be expected from the use of the long forceps, employed by the most expert operators, who are not always to be obtained in these emergencies. Certainly for ordinary practitioners to resort to this instrument under such circumstances would be highly improper, to use the mildest terms, and is even deprecated by both author and translator of this work a few pages on: so that the true drift of his recommendations is somewhat difficult to arrive at. Dr. Chailly says " when the head is half engaged in the superior strait, the rules for the application of the forceps are precisely the same as when it has descended into the cavity. The operation, however, is more difficult and dangerous for both mother and child. It should, therefore, never be had recourse to, except where version is impracticable in consequence of the excessive ascent of the uterus, and (is it possible he can recommend its employment in place of the perforator in this case?) when the pelvis is malformed or the size of the head very large. The regular application of the branches at the superior strait is exceedingly difficult: *indeed it may be said to be impossible in most cases.*" Let the reader bear in mind these are the words of one of the most expert of French accoucheurs, kept in constant practice by the resources of a large hospital; and then ask himself whether, in general practice, the risking the safety of the mother for the very remote chance, under these circumstances, of preserving the life of the child, is a justifiable procedure. " Too much attention," says the translator, " cannot be given to the opinion of M. Chailly in reference to the *difficulty and danger of applying the forceps where the head is at the superior strait* * * * * * a rule which I hold to be almost universally proper: to turn rather than attempt delivery by the forceps when the head is still at the superior strait."

We quite agree however with this gentleman in deprecating the practice of MM. Dubois and Chailly of employing the forceps in a *furtive* manner. " Keep the patient in ignorance," they say, " of what you are about to do, assure her that the hand alone will suffice, conceal the instrument from her sight, and be careful not to make any noise in locking the branches. With this view the forceps should be enveloped in a cloth, and placed on the floor at the foot of the bed." We can, indeed, conceive the case of some of these young French accoucheurs, to whom this advice is addressed, being hereafter not only very desirous of concealing that they were about to employ this instrument, but also of *preventing its being known that they had done so.* In England, we believe, that no lecturer would recommend, or any practitioner attempt, to employ this, or any other surgical instrument, independently of the patient's entire knowledge of the object in view, and free consent to the endeavour at its accomplishment.

In another point also we cordially agree with Dr. Bedford, namely, his recommendation of the more frequent employment of the *manakin.* The majority of lecturers and great body of pupils neglect this far too much; for, although the most valuable lesson that can be taught the pupil is a full reliance upon the great powers of Nature, and the impropriety of hastily interfering with her proceedings, yet the acquisition of sufficient dexterity for the employment of interference when it is necessary is of scarcely less importance. Nay its possession is available in even the common routine of midwifery operations; and many imaginary difficulties in the extraction of the placenta (some of which are recorded as rare cases) have arisen from the untaught clumsiness of the operator.

"I cannot too strongly impress upon the pupil the value of this machine. To become a dexterous and safe obstetric operator, two things are necessary, and they are so essentially material that, if they be neglected, failure must often be the result. In the first place, the accoucheur should have a clear perception of the principles on which rests the mechanism of natural labour. And, secondly, he should reduce those principles to practice on the manakin. The manakin is as important to the obstetrician as the cadaver to the surgical operative surgeon: and if practitioners could be made to appreciate the benefits to be derived from practising on it, much good would necessarily result to those, who in their hour of peril, need not only the sympathy, but the consummate skill of their medical attendant." 320.

The author makes no allusion whatever to the lever or tractor.

